# Guarding Against Catastrophic Biological Risks: Preventing State Biological Weapon Development and Use by Shaping Intentions

**DOI:** 10.1089/hs.2022.0145

**Published:** 2023-07-18

**Authors:** Jaime M. Yassif, Shayna Korol, Angela Kane

**Affiliations:** Jaime M. Yassif, PhD, is Vice President, Nuclear Threat Initiative, Washington, DC.; Shayna Korol is Biosecurity Policy Fellow, Global Biological Policy and Programs, Nuclear Threat Initiative, Washington, DC.; Angela Kane, MA, is Senior Advisor; all at the Nuclear Threat Initiative, Washington, DC.

**Keywords:** COVID-19, Biological Weapons Convention (BWC), International coordination, Catastrophic risk, Building security

## Background

The devastating impact of COVID-19 has highlighted global vulnerabilities to high-consequence biological events. The international community was woefully unprepared for a pandemic that has led to millions of deaths and trillions of dollars in economic losses, and has upended daily life. However, notwithstanding the severe damage caused by COVID-19, it should be viewed as a warning shot.^1^ It will not be the last pandemic humanity faces, and the next high-consequence biological event could be as destructive or substantially worse.

We define global catastrophic biological risks (GCBRs) as biological events of tremendous scale that could cause severe damage to human civilization, potentially jeopardizing its long-term survival.^2^ The Johns Hopkins Center for Health Security has also developed a working definition of GCBRs,^3^ and this term is part of a broader discussion about global catastrophic risks that could arise from a variety of sources, including nuclear war, anthropogenic climate change, and advanced artificial intelligence that has not been sufficiently safeguarded.^4,5^ GCBRs could be caused by a naturally emerging infectious disease outbreak, an accidental release of a pathogen, or a deliberate attack. Naturally emerging infectious disease outbreaks that can grow into pandemics are likely to increase in frequency due to urbanization, globalization, and environmental degradation, and the world faces an increasing risk of high-consequence biological events resulting from accidental or deliberate misuse of the tools of modern bioscience and biotechnology.^6-8^ Not all outbreaks or global pandemics will grow to the scale of a GCBR as we define it in this article and as others have defined global catastrophic risks more broadly, because the threshold for this type of event is extremely high.

Although COVID-19 does not rise to the level of a GCBR-scale event, it has demonstrated that a biological event can have a devastating global impact, and it should serve as a warning to global leaders that the world needs much more robust protections against high-consequence biological events that could emerge in the future and be substantially worse.

In our view, human-caused biological events involving the accidental or deliberate misuse of an engineered pathogen are more likely to lead to a GCBR-scale event than a naturally emerging pandemic.^9^ Scientists have the capacity to deliberately or inadvertently engineer pathogens that are more virulent and transmissible than what nature creates by chance, and the upper limit of damage that could be caused by a human-engineered biological event is unknown.^10-12^ Prevention, early detection, and rapid response are all crucial for guarding against GCBR-scale events. However, in this article, we focus on effective strategies for preventing biological events that could become GCBRs, specifically by disincentivizing development and use of biological weapons by states and other powerful actors.

Work to prevent the development and use of biological weapons is crucial. While biotechnology advances offer tremendous potential benefits—including improvements in public health, economic development, and climate change—rapidly advancing capabilities to manipulate biological systems are also making it easier to engineer increasingly sophisticated biological weapons.^5,13^ These advances are making it possible for a wider range of actors to exploit biology to cause catastrophic harm. Unfortunately, the devastation caused by COVID-19 may have exacerbated this vulnerability by making biological weapons more attractive as a means to achieve political, economic, or other more radical objectives.^14^

Two types of actors could pose important risks to bioweapons development and use: states and nonstate actors. Any effective strategy for countering these risks will ultimately need to reduce the likelihood that an actor will have both the capability and intention to develop and/or use a biological weapon that could cause a GCBR-scale event.

To prevent the development and use of biological weapons by nonstate actors, the most effective strategy is to constrain their capabilities to cause catastrophic harm. Nonstate actors are not typically motivated by the same rational economic, political, and military goals that incentivize states, and they may have an apocalyptic vision aligned with using biological weapons to cause global catastrophic harm. Lawrence Kerr has noted that “at one point in time, there were 3,000 named apocalyptic groups around the world,” including terrorists “solely interested in annihilation of humans.”^15^ It is fair to assume that some of these groups would readily use biology to cause globally catastrophic harm if given the opportunity.^16^ Aum Shinrikyo is an example of an apocalyptic cult that pursued the development of chemical and biological weapons, making several attempts to carry out large-scale biological attacks in Japan in the 1990s; fortunately they were unsuccessful.^17^ Importantly, nonstate actors usually lack the resources of states, especially in terms of trained personnel and financial assets, which makes it feasible to constrain their capabilities by denying them access to materials, equipment, and the technical expertise needed to develop or acquire biological weapons. This strategy can be achieved by safeguarding the tools of modern bioscience and biotechnology to prevent their exploitation by malicious actors.^18-21^

In contrast, many nation states have substantial financial resources at their disposal and access to trained personnel, which makes it extremely difficult and perhaps even impossible to constrain their capabilities to develop and use powerful biological weapons. In our view, a much more effective strategy for preventing the development and use of biological weapons by states is to make the potential costs of bioweapons development unacceptably high and to diminish any perceived benefits.^9^ Although some states may see potential tactical or strategic benefits of developing biological weapons—or even using them in some cases—they generally have rational political, economic, and military objectives that would not be well served by deliberately causing a GCBR. Nevertheless, bioweapons development can have unintended consequences—including accidental release of a highly transmissible, deadly biological agent—and any attempt at targeted bioweapons use could result in a much larger and more widespread biological event than intended.^22^ For these reasons, we contend that state biological weapons programs can and should be countered with well-designed incentive structures.^23^ Designing and building such structures is the focus of this article.

## States, Biological Weapons, and GCBRs

To develop effective disincentives for bioweapons development or use, it is important to first understand the range of possible motives for states to consider such weapons. First, misperceptions or suspicions regarding other countries' bioscience capabilities and intentions, exacerbated by a lack of transparency, can drive arms-racing dynamics. Second, states may decide that bioweapons are potentially useful for tactical purposes—for example, to carry out a covert, plausibly deniable economic attack, or if they believe they could carry out a targeted attack that did not impact their own population or that of their allies.^24,25^ Third, states may be interested in bioweapons as a strategic weapon for deterrence, potentially as a more accessible and affordable option than developing nuclear weapons.^26^ These tactical and strategic incentives could grow over time as geopolitical tensions continue to escalate between the United States, China, and Russia—and as relationships among regional powers face growing strains.^22^ Additionally, even if political leadership does not set bioweapons development as a goal, bureaucratic forces and perverse incentives within large government organizations can drive development.^27^ Advances in science and technology that make it easier to engineer living systems could influence all of these considerations and shape states' cost-benefit analyses regarding the potential utility of bioweapons. Addressing the risk is not a hypothetical challenge, as there is evidence that several states currently possess bioweapons programs,^28^ and many more have the latent capability to pursue such programs if they choose to do so.

## Gaps in the Current Biosecurity Architecture

The need to guard against state bioweapons programs is crucial and growing, for the reasons previously outlined, but the global biosecurity architecture lacks adequate mechanisms and resources to disincentivize and deter the development and use of these weapons. First, while the Biological and Toxin Weapons Convention (BWC) is essential for upholding the norm against the development and use of biological weapons, it is woefully underresourced. With an annual budget of US$1.5 million, the BWC lacks the financial resources to fulfill its mandate to effectively prohibit “the development, production, acquisition, transfer, stockpiling and use of biological and toxin weapons.”^29,30^ Importantly, unlike the Chemical Weapons Convention and the Nuclear Non-Proliferation Treaty,^31^ the BWC lacks an associated operational organization^32^ and currently has only an Implementation Support Unit with 3 full-time staff members.^33^

The BWC also lacks adequate transparency measures to assess and assure compliance. While it has Confidence Building Measures (CBMs), established in 1986 and designed to increase transparency,^34^ the tool is insufficient to reduce suspicions about other nations' dual-use bioscience research and development activities. In addition to suffering from a low participation rate, the CBM form itself is outdated and inappropriate for today's advanced global bioscience and biotechnology research and development enterprise. Furthermore, there is no defined process for follow-up or assessment of the information shared by states. Many experts also have lamented the absence of a BWC verification regime.^14,35^ Although there is no consensus within the biosecurity community that verification is practically achievable, our view is that more robust transparency measures that far exceed the scope of CBMs are needed. Without such measures, substantial gaps in the BWC will remain.

Although the global biosecurity architecture includes additional mechanisms outside of the BWC—such as UN Security Council Resolution 1540,^36^ the Australia Group,^37^ and the 1925 Geneva Protocol^38^—none of these address the gaps outlined in this section. UN Security Council Resolution 1540 is primarily a tool for states to prevent weapons of mass destruction terrorism, including bioterrorism; the Australia Group export control regime is primarily a means of constraining capabilities, which as previously discussed, is a weak measure for preventing bioweapons development by states; and the provisions of the Geneva Protocol, which bans the use of biological weapons, have effectively been incorporated into the BWC.^29^

Addressing key gaps in the global biosecurity architecture will be difficult, especially in the current geopolitical environment, because of the consensus-based decisionmaking approach currently used by BWC states parties that enables a single state to derail constructive dialogue and progress. To close gaps within the BWC and across the broader biosecurity architecture, new and innovative approaches that build stronger systems around the BWC and establish legitimacy through a variety of channels will be necessary.

## A New Approach to Strengthening International Capabilities to Prevent Biological Weapons Development and Use

To address the gaps previously discussed, we outline an agenda that revolves around effectively shaping the cost-benefit calculation of states to make biological weapons an unattractive option. We envision 3 key elements of a new strategy to shape state intentions: (1) enhanced transparency and BWC compliance assurance, (2) more robust capabilities to assess and attribute the origins of biological events, and (3) a well-defined system of accountability for BWC violations.

### Enhancing Transparency

Transparency is critical for reducing the potential appeal of bioweapons development. Effective transparency measures can help avoid misperceptions and unwarranted suspicions regarding other nations' bioscience and biotechnology activities, which could otherwise drive arms-racing dynamics. Enhanced transparency measures can provide greater assurances regarding BWC compliance, and in rare instances, such measures may be able to detect violations of the BWC.^39^

At present, CBMs are the primary official transparency measure under the BWC, and there are near-term opportunities to incrementally improve them. These opportunities include updating CBM forms to make them more rigorous and appropriate for modern bioscience and biotechnology, equipping the BWC Implementation Support Unit with resources to analyze the content of CBM submissions, and providing training for countries to prepare CBMs to increase the participation rate, which is barely above 50%.^40^ However, while helpful, such reforms would constitute incremental progress where more fundamental transformation is needed.

There is currently a political opening to discuss a more ambitious approach to building robust transparency measures, due in part to a statement by the US delegation at the 2021 BWC Meeting of States Parties. Ambassador Bonnie Jenkins, who delivered the statement, argued that the upcoming BWC Review Conference “should establish a new expert working group to examine possible measures to strengthen implementation of the Convention, increase transparency, and enhance assurance of compliance.”^41^ This statement led to renewed discussions about potentially viable approaches to BWC verification—an issue that did not have broad support across states parties since negotiations on a verification protocol fell apart in 2001, and which was previously opposed by the United States.^42^ The subsequent 2022 BWC Review Conference successfully established a working group that will discuss measures on confidence building and transparency and measures on compliance and verification.^43^ Recent efforts to explore new approaches to verification include a report on the topic by the United Nations Institute for Disarmament Research, a Wilton Park workshop cohosted by the iGEM Foundation, and the 2022 Next Generation for Biosecurity Competition on the topic.^44-46^ Innovative thinking from a new generation of experts is crucial for advancing meaningful approaches to transparency and exploring the possibility of BWC verification, and these efforts represent a promising step in this direction.

It is important to acknowledge uncertainty as to whether a full verification regime for the BWC is technically feasible. A key issue is that bioscience research is deeply dual use, so even with intrusive inspections that reveal important details about the pathogens facilities are working with, such information will not necessarily be sufficient to determine whether bioweapons development is underway.^47^

Even if full verification of compliance is not achievable, introducing “enhanced transparency measures”^48^—including many of the same components of a verification regime but without the high confidence assessments of compliance or lack thereof—would strengthen the BWC. One potentially promising approach is to build on existing voluntary peer review visits, in which a number of governments have conducted site visits to each other's facilities to bolster transparency.^49,50^ Institutions could be encouraged to voluntarily undergo more detailed assessments to demonstrate BWC compliance; incentives could include priority regulatory review, special access to funding, and reduced insurance premiums, among other options.^45^

In addition to efforts by governments, enhanced transparency measures can involve a broader range of stakeholders, drawing on expertise from the biotechnology industry and the academic research community. Ongoing scientific and technological advances continue to transform what is possible for both onsite and offsite assessments of bioscience research facility activities. Leaders from industry, academia, and civil society have an opportunity to develop and run pilot projects on enhanced transparency measures to explore what is possible with the goal of validating a range of new approaches for conducting assessments of bioscience research facilities. A crucial part of these pilot projects will be to find ways to conduct rigorous onsite and offsite assessments while protecting the intellectual property of industry and academic research facilities.

### Strengthening Attribution

Attribution is another crucial pillar in disincentivizing bioweapons use. States must believe that there is a considerable chance they will be caught if they use or accidentally release biological weapons. To accomplish this, the international community needs stronger capabilities to assess the origins of high-consequence biological events.

This includes strengthening existing mechanisms, especially the United Nations Secretary-General's Mechanism (UNSGM) for Investigation of Alleged Use of Chemical and Biological Weapons.^51^ The mechanism is not a standing investigative body, but it relies on a roster of qualified experts, laboratories, and expert consultants nominated by member states who can be called upon to support a UNSGM investigation under short notice. Although the UNSGM is not part of the BWC, it plays an important role in supporting and strengthening it. It is welcome news that the operational capabilities of the UNSGM to investigate bioweapons allegations have continued to advance, but there is still room for improvement. To start with, the UNSGM needs substantially more financial resources to be truly effective.^52,53^ It is currently supported through voluntary, in-kind contributions by a group of member states known as the “Friends of the UNSGM,” but it could benefit from a wider range of supporters, including contributions from an expanded roster of UN member states as well as philanthropic donors.

In addition to strengthening the UNSGM, it will also be important to fill gaps. As evidenced by early challenges with discerning COVID-19 origins, current international mechanisms are insufficient to discern the source of ambiguous biological events. The UNSGM has the mandate to investigate allegations of deliberate bioweapons use, but the bar for triggering an investigation is high; it can only be triggered by the secretary-general in response to the request of a member state.^54^ In practice, a high standard of evidence is needed to credibly make this type of claim and for the secretary-general to follow through by launching the UNSGM. To date, the UNSGM has been activated 3 times to investigate allegations of chemical weapons use,^55^ but it has never been activated to investigate alleged bioweapons use.

In most cases, the World Health Organization would provide the initial international response to a biological event and would be the first organization collecting information on the ground. Although the World Health Organization has authority under the International Health Regulations (2005) to respond to biological events regardless of origin, its key operational strength, and the comfort zone of its member states, is its ability to assess and respond to naturally emerging infectious disease outbreaks. It is unclear how far the World Health Organization would be willing or able to go in assessing the origins of accidents or biological weapons attacks. These limitations mean that investigating high-consequence biological events of unknown origin falls between current mechanisms.^56^

To fill this gap, the Nuclear Threat Initiative proposed a new Joint Assessment Mechanism for discerning the source of high-consequence biological events of unknown origin that would build on existing capabilities and mechanisms with the aim of creating an integrated UN approach to assessing pandemic origins.^57^ It would not be part of the BWC but could strengthen the broader biosecurity architecture that supports it. The proposed Joint Assessment Mechanism would be based within the UN secretary-general's office and established under their authority. It would include a standing capability with a small team responsible for integrating and analyzing data on an ongoing basis, and the ability to rapidly launch an assessment when triggered by the UN secretary-general.

This proposal is part of a broader discussion about ambitious proposals to restructure the international biosecurity architecture. Ambassador Ahmet Üzümcü, former director-general of the Organisation for the Prohibition of Chemical Weapons, put forward a proposal to establish the International Biotech Organization. The International Biotech Organization's core mission would be to “rapidly deploy its technical experts to simultaneously identify the pathogen behind an emerging outbreak while providing public health measures and medical advice to the local authorities on the ground.” The independent organization would engage both public and private stakeholders in the biotechnology industry and scientific and philanthropic communities, and would address pathogens that may be accidental, deliberate, or natural in origin.^58^

If these types of mechanisms had existed in 2019 or 2020, some of the difficulties in identifying COVID-19's origins and mitigating its effects might have been avoided.^20^ Stronger tools for attribution can have profound implications for international security: for example, determining that a high-consequence biological event is naturally occurring can allay suspicions about BWC violations. Stronger tools for attribution may deter powerful actors from developing and using biological weapons by increasing the likelihood that they would get caught in the act.^59^

### Building an Accountability System

In addition to believing that they are likely to be caught if they develop or use biological weapons, for deterrence to be effective, states must also believe that they will be held accountable. Yet, there is currently no defined international mechanism for accountability in the event of bioweapons development or use.

Failures of accountability following well-documented chemical weapons use have undermined norms enshrined in the Chemical Weapons Convention and illustrate the substantial challenge that the BWC would face if presented with an analogous situation. For example, the mandate of the Organization for the Prohibition of Chemical Weapons-UN Joint Investigative Mechanism, which was created by the UN Security Council to identify and hold accountable those responsible for chemical weapons attacks in Syria, lapsed when Russia voted against renewing its mandate.^31,60^ Additional examples include the use of the Novichok nerve agent in an assassination attempt of Sergei Skripal in Salisbury, England, and to poison Russian opposition leader Alexei Navalny. In both cases, Russia denied responsibility, and the perpetrators have not been brought to justice.^61-63^ North Korean leader Kim Jong Un's estranged half-brother Kim Jong Nam was assassinated with the chemical warfare agent VX in Malaysia; North Korea denied responsibility for the attack, and charges against the suspects were dropped.^64,65^

To avoid erosion of the norm against bioweapons development, it will be essential to establish a clearly defined accountability system for any BWC violations that may arise. Under the UN system and multilateral treaties that rely on consensus, attempts to hold states accountable often become politicized discussions that depend on the geopolitical status and relationships of the state in question. This is a challenge without a clear or easy solution, and policymakers need to explore accountability approaches that cannot be voted down by a single state or powerful coalition. The international community can employ a broad set of tools, including economic sanctions, political pressure and isolation, and in the most extreme cases, military action. Proportionality will be important in deploying these responses, and it would be valuable to lay out a road map for which types of responses are warranted under a range of circumstances.

The biosecurity community can learn from analogous efforts to demand accountability for chemical weapons development and use. For example, at least 27 countries expelled Russian diplomats in the wake of the Salisbury Novichok poisonings. Such international, coalition-based responses send a firm message about the importance of respecting global norms against illicit use of unconventional weapons.^66^ In the event of a BWC violation, states parties should coordinate their responses as much as possible to demonstrate shared political will and a united front in demanding accountability. To make this type of response possible, individual countries need to be willing to take action in the face of bioweapons development or use.^22^

The international community must adopt a zero-tolerance policy in response to confirmed cases of biological weapons development or use. To operationalize this, one promising approach could be to develop a biosecurity analogue to the International Partnership Against Impunity for the Use of Chemical Weapons. This intergovernmental initiative, composed of 40 states and the European Union, supports multilateral action to hold perpetrators accountable by compiling and sharing information on those involved in chemical weapons use.^67,68^

## Conclusion

The world is vulnerable to GCBRs, and those risks are growing over time. New approaches to disincentivize states from developing or using bioweapons have the potential to be highly effective and are crucial for strengthening global biosecurity and preventing GCBRs. The COVID-19 pandemic has highlighted our shared vulnerabilities and provided an important reminder of the urgent need to strengthen our defenses against global biological catastrophes. At the same time, growing geopolitical tensions, accompanied by increasingly aggressive disinformation campaigns and false allegations regarding biological weapons, put the BWC and the broader global biosecurity architecture under strain. We must leverage the opportunity to tackle the challenges of enhancing transparency, improving attribution, and fostering accountability for violating the global norm against bioweapons development and use. If the biosecurity and broader international community can come together to develop robust measures for ensuring BWC compliance, we can create a safer, more sustainable future for generations to come.
